# Factors Affecting the Prevalence of Strongly and Weakly Carcinogenic and Lower-Risk Human Papillomaviruses in Anal Specimens in a Cohort of Men Who Have Sex with Men (MSM)

**DOI:** 10.1371/journal.pone.0079492

**Published:** 2013-11-20

**Authors:** Dorothy J. Wiley, Xiuhong Li, Hilary Hsu, Eric C. Seaberg, Ross D. Cranston, Stephen Young, Gypsyamber D’Souza, Otoniel Martínez-Maza, Katherine DeAzambuja, Kristofer Chua, Shehnaz K. Hussain, Roger Detels

**Affiliations:** 1 School of Nursing, University of California Los Angeles, Los Angeles, California, United States of America; 2 Bloomberg School of Public Health, Johns Hopkins University, Baltimore, Maryland, United States of America; 3 School of Medicine, University of Pittsburgh, Pittsburgh, Pennsylvania, United States of America; 4 Tricore Diagnostic Laboratories, University of New Mexico, Albuquerque, New Mexico, United States of America; 5 David Geffen School of Medicine at University of California Los Angeles, Los Angeles, California, United States of America; 6 UCLA AIDS Institute, University of California Los Angeles, Los Angeles, California, United States of America; 7 Jonathan and Karen Fielding School of Public Health, University of California Los Angeles, Los Angeles, California, United States of America; University of Cape Town, South Africa

## Abstract

**Background:**

MSM are at higher risk for invasive anal cancer. Twelve human papillomaviruses (HPVs) cause cervical cancer in women (Group 1 high-risk HPVs (hrHPVs)) and 13 HPVs are probable/possible causes (Group 2 hrHPVs) of cervical malignancy. HPVs rarely associated with malignancy are classified as lower-risk HPVs (lrHPVs).

**Materials and Methods:**

Dacron-swab anal-cytology specimens were collected from and data complete for 97% (1262/1296) of Multicenter AIDS Cohort Study (MACS) men tested for HPVs using the Linear Array assay. Multivariate Poisson regression analyses estimated adjusted prevalence ratios for Group 1/2 hrHPVs and lrHPVs, controlling for the effects of age, race, ethnicity, sexual partnerships, smoking; HIV-infection characteristics, treatment, and immune status among HIV-infected men.

**Results:**

HIV-infected men showed 35–90% higher prevalence of Group 1/2 hrHPVs and lrHPVs than HIV-uninfected men, and higher prevalence of multi-Type, and multiple risk-group infections. CD4+ T-cell count was inversely associated with HPV Group 2 prevalence (p<0.0001). The number of receptive anal intercourse (RAI) partners reported in the 24 months preceding HPV testing predicted higher prevalence of Group 1/2 hrHPVs. Men reporting ≥30 lifetime male sex partners before their first MACS visit and men reporting ≥1 RAI partners during the 24 months before HPV testing showed 17–24% and 13–17% higher prevalence of lrHPVs (*p*-values ≤0.05). Men reporting smoking between MACS visit 1 and 24 months before HPV testing showed 1.2-fold higher prevalence of Group 2 hrHPVs (p = 0.03). Both complete adherence to CART (*p* = 0.02) and HIV load <50 copies/mL (*p* = 0.04) were protective for Group 1 hrHPVs among HIV-infected men.

**Conclusions:**

HIV-infected men more often show multi-type and multi-group HPV infections HIV-uninfected men. Long-term mutual monogamy and smoking cessation, generally, and CART-adherence that promotes (HIV) viremia control and prevents immunosuppression, specifically among HIV-infected MSM, are important prevention strategies for HPV infections that are relevant to anal cancer.

## Introduction

Invasive anal cancer (IAC) is a health crisis for gay, bisexual, and other men who have sex with men (MSM). Overall, IAC rates have increased steadily among U.S. men between 1975–2008, but risk among HIV-infected MSM has increased greatly since the introduction of combined antiretroviral therapy (CART) for HIV infection in the late 1990 s [1]–[5]. Current domestic IAC incidence estimates among MSM surpass contemporary invasive cervical cancer rates in women as well as rates reported when domestic mass cervical cytology screening was initiated in the 1950 s [2], [4], [6]–[11]. Currently, domestic IAC incidence rates among HIV-infected MSM surpass rates for 7 of the top 10 cancers, 13–36 cases/100,000, and even exceeds the third most common cancer, colorectal cancer, for all U.S. males: conservatively, 78 vs. 53/100,000, respectively [1], [2], [4], [12], [13].

Human papillomaviruses (HPVs) infect epithelial cells and show varying degrees of pathogenicity from warts to invasive cancers. Until recently, 14 viruses were referred to as high-risk HPVs (hrHPVs): HPV16, 18, 31, 33, 35, 39, 45, 51, 52, 56, 58, 59, 68, and 69 [14], [15]. Currently, the first 12 are classified as Group 1 hrHPVs, and considered necessary but insufficient causes of cervical cancer by the International Agency for Research on Cancer (IARC) [14]. Among these, HPV16 is considered most pathogenic and is detected most often in cancers [14]. Experts generally agree that most Group 1 hrHPVs are strong carcinogens [16]. Additionally, IARC recently regrouped HPV68 and 69, together with 11 other viruses, HPV26, 30, 34, 53, 66, 67, 70, 73, 82, 85, and 97 as *probable* or *possible*, weaker carcinogens, collectively evaluated as Group 2 hrHPVs [14], [16]. Low-grade dysplasias, such as genital warts, are commonly caused by HPVs that are seldomly associated with cancers. Lower-risk HPV (lrHPV) Types 6, 11, 40, 42, 54, 55, 61, 62, 64, 71, 72, 81, 83, 84, Is39, and CP6108 can be simultaneously characterized with Group 1 and 2 hrHPVs using PCR and dot-blot hybridization (HPV-Linear Array (HPV-LA), Roche Diagnostics, Pleasanton, CA) [17]–[19]. However, the HPV-LA assay only evaluates HPV26, 53, 66, 67, 68, 69, 70, 73, and 82 Group 2 hrHPVs, but not HPV30, 34, 85, and 97 [17]–[19].

Anal-genital HPV prevalence estimates for men vary widely, over time and across studies, and methodological differences make it difficult to compare findings across these groups [20]–. No published study evaluates the HPV-infection characteristics in a well-described population of HIV-infected and -uninfected MSM 50 years or older who are likely to be at highest risk for anal cancer [2], [20], [23], [24], and little is known about the effect of HIV-treatment adherence on HPV infection in affected MSM [25]. Additionally, no studies yet evaluate sociodemographic and behavioral risk factors for Group 1 and 2 hrHPVs separately using International Agency for Research on Cancer (IARC) risk groupings [14]. Accordingly, data for 1,262 well-described Multicenter AIDS Cohort Study (MACS) participants were evaluated to test the hypotheses that age, and other social and demographic characteristics, and longitudinally-reported sexual behaviors positively affected Group 1 and 2 hrHPV and lrHPV prevalence, while HIV-treatment and some –infection characteristics differentially influence the prevalence of hr- and lrHPVs.

## Materials and Methods

### Subjects and Setting

The Anal Health Study was approved locally at each of 4 U.S. MACS study sites: IRB#:84-03-02-01-1 (Baltimore) approved by the Johns Hopkins Bloomberg School of Public Health Institutional Review Board (IRB), 01–225/CR3-STU00022906 (Chicago) approved by the Northwestern University IRB, 10-001677-CR-00002/103360-11 (Los Angeles) approved by the UCLA IRB, and REN12070132/IRB9505118 (Pittsburgh) approved by the University of Pittsburgh IRB. Men were invited by research personnel to participate in the AHS at the first MACS visit following the study’s opening in May 2010. Written informed consent for anal Pap test and HPV genotyping was obtained and study specimens were collected for 1,296 MSM at the first AHS study visit completed between May 2010 and February 2011. Of these, data were complete and HPV assays were interpretable for 97.3% (1262/1296). The MACS is described extensively elsewhere [26]–[28]. Briefly, 2,291 men were in active follow-up as of March 31, 2012∶61% (1389/2291) were enrolled before 2001 and 49% (1131/2291) were HIV-infected. Since the mid-1990 s, the study aims focus on describing the natural history of HIV-infection in the treatment era [26]. MACS participants are examined semi-annually using standard protocols by trained examiners and demographic, general health, neuro-cognitive, sexual and behavioral characteristics are collected using both interviewer-guided and self-administered questionnaires. Laboratory specimens are gathered and preserved or tested semi-annually using standard MACS protocols; for example, only sera for men testing negative at the prior study visit are evaluated for HIV antibody using enzyme-lined immunosorbant assays and western blot [27].

### Exposures of Interest

Demographic, sexual, behavioral, and HIV-infection characteristics and HIV-related laboratory estimates for CD4+ T-lymphocyte counts were evaluated. Data for Black, Asian, American Indian or Alaskan Native, and other races (non-Whites), were compared to those of Whites; men reporting Hispanic ethnicity were compared to non-Hispanics. Sexual exposures were evaluated using three different measures: 1) the number of lifetime male sex partners reported at the first MACS visit (>300, 100–299, 30–99, vs. <30), 2) the number of male sex partners reported at each semi-annual study visit between the first MACS visit and the study visit 24 months before HPV testing (>200, 100–199, 30–99 vs. <30), and 3) the number of male receptive anal intercourse (RAI) partners reported at each of the last four semi-annual visits preceding the HPV-test visit (≥4, 1–3, vs. 0). Men who reported smoking (tobacco) were compared to never-smokers for two periods: 1) MACS visit 1 to 24 months before HPV testing and 2) over the last 24 months before testing. Data for HIV-infected men with >500, 350–500 and <350 CD4+ T-cells/mm^3^ at the HPV test-visit were compared to those of HIV-uninfected men; for analyses of HIV-infected men alone, those with >500 CD4+ T-cells/mm^3^ served as the referent group. HIV-infection duration was estimated as the total number of years since enrollment for prevalent positive men and since HIV-antibodies were detected for HIV-seroconverters. Multiple measures of HIV-infection control evaluated optimal versus less optimal HIV-infection treatment and control: 1) the proportion of total visits where HIV mRNA (HIV-load) measured ≤50 copies/mL in serum (Roche Amplicor 2.0, Roche Molecular Inc., Pleasanton, California), 2) the lowest CD4+ T-cell measurement measured since HIV was first detected by the MACS (<200 vs. ≥200 cells/mm^3^), 3) the number of years of CART treatment (per year), and 4) self-reported complete adherence to CART over the 4 days preceding the HPV-test visit (yes/no). Data for three study sites were compared to the largest AHS-enrollment site and data for men recruited before 2001 were compared to men entering thereafter to control for the effects of geographic variability and time.

### Outcome of Interest

The prevalence of Group 1 and 2 hrHPVs and lrHPVs were evaluated using residual anal cytology specimens and PCR. Briefly, anal cytology specimens were collected using a standard protocol: Dacron swabs were inserted ∼5 cm beyond the anal verge, approximated to the anal wall, rotated slowly while being withdrawn to capture cells and pathogens, and placed into preservative (PreservCyt, Cytyc Corporation, Boxborough, MA). DNA was extracted from residual cytology specimens (AmpiLute Media Extraction Kit, Qiagen, Valencia, CA) by a single laboratory (Tricore Reference Laboratory, Albuquerque, New Mexico). The HPV-LA uses the PGMY09/PGMY11 primers to characterize the L1 consensus region of HPV DNA (Roche Diagnostics, Pleasanton, California) [17]. Two concentrations of a β-globin control probe assessed specimen adequacy and PCR inhibition. The assay detects up to 37 targeted HPV types with 94.8% sensitivity and 38% specificity for predicting cytological disease, especially high-grade cervical intraepithelial neoplasia and invasive cervical cancer [29]. HPV-LA shows high concordance with findings from other HPV genotyping assays (Kappa = 0.78, p<0.001) [17], [29], [30]. Analyses compared group-specific prevalence estimates for hr- and lrHPVs: Group 1, strongly carcinogenic hrHPVs, HPV16, 18, 31, 33, 35, 39, 45, 51, 52, 56, 58, and 59; Group 2, weakly carcinogenic hrHPVs, HPV26, 53, 66, 67, 68, 69, 70, 73, and 82 (not 30, 34, 85, 97); and lrHPVs, HPV6, 11, 40, 42, 54, 55, 61, 62, 64, 71, 72, 81, 83, 84, Is39, CP6108 [14].

### Statistical Analyses

Descriptive and tabular analyses were used to explore the data. The Fisher exact or the Wilcoxon nonparametric tests, as appropriate, were used to test differences in proportions and distributions of characteristics for HIV-infected and HIV-uninfected men. For age-group comparisons of prevalence estimates, the exact 95% confidence intervals were estimated using the binomial distribution. Poisson regression with robust error variance [31] analyses were used to estimate prevalence ratios (PR) and 95% confidence intervals (CI) for Group 1 and 2 hrHPVs, and lrHPVs, comparing demographic, sexual behavioral characteristics, HIV-infection characteristics, and recruitment period in both univariate and multivariate models using the SAS 9.2 GENMOD procedure (SAS Institute, Cary, North Carolina, United States of America). All models included age, race, period-specific self-reported number of sexual partnerships, HIV-infection characteristics, and tobacco-smoking. Additionally, comparisons among HIV-infected men alone also included HIV-infection duration and -load and -treatment features, and CD4+ count (cell/mm^3^) characteristics at the HPV-test visit and the lowest measurement observed across the observation period.

## Results

### Descriptive Analyses

The 1,262 participants were predominantly older, White MSM of whom nearly 80% (1005/1262) tested positive for ≥1 HPVs. On average, men were 55 (±9.4) years old at the HPV-test visit, and nearly 68% (859/1262) had joined the study in 2001. Hispanic ethnicity was reported by 8% (100/1262), and self-report for race showed 79% (993/1262) were White, and 21% were minority races (Table 1). Nearly half (46%, 579/1262) were HIV-infected. However, minority men showed 1.6-fold higher prevalence of HIV-infection than White participants: 65% (59%, 70%) vs. 41% (38%, 44%), respectively (p<0.001). Only 16% (93/579) of HIV-infected men reported *any* prior AIDS-defining illnesses at the first HPV test visit and while HIV-infected men showed lower CD4+ T-cell counts than -uninfected men, their levels measured high, on average, at the HPV-test visit: 609 cells/mm^3^ vs. 929 cells/mm^3^, respectively (*p*<0.05) (Table 1). Over the entire study period, HIV-infected men showed HIV-load measurements <50 copies/mL at ∼53% of visits; however, the nadir CD4+ T-cell count observed among these men, on average, was 258 cell/mm^3^ (Table 1). Smoking most dramatically changed over the course of the MACS study among HIV-uninfected men. The majority of participants (72%; 913/1262) reported smoking at least once between the first MACS visit and the visit 24 months before HPV testing, HIV-infected men were 1.8-times more likely to report smoking than –uninfected men during the last 24 months before HPV testing (Table 1). The number of lifetime male sex partners reported at the first MACS visit varied widely and the distribution was skewed right: µ = 270 (±488), median (M) = 100), range: 1–9999+, and 29% (349/1222) reported ≥300 lifetime partners. However, nearly 74% (920/1250) of men reported 0–3 RAI partnerships during the last 24 months before HPV testing. Overall, HIV-infected men showed 30% higher prevalence of HPV infection than HIV-uninfected men (PR = 1.3 (1.2, 1.4), prevalence: 91% (525/579) vs. 70% (480/683), respectively; Table 1)).

**Table 1:** Sociodemographic Characteristics, Sexual Behavior Characteristics, HIV, and Human papillomavirus (HPV) infection Characteristics of 1262 Multicenter AIDS Cohort Study Participants Evaluated for HPV DNA using Anal Swab Specimens.

	HIV Seropositive	HIV Seronegative	Total
	(N = 579)	(N = 683)	(N = 1262)
	n (%)	n (%)	n (%)
**Characteristic**			
**Age (years)**			
<30	4 (01)	9 (01)	13 (01)
30–39	31 (05)	34 (05)	65 (05)
40–49	154 (27)	108 (16)	262 (21)
50–59	280 (48)	277 (41)	557 (44)
60–69	101 (17)	203 (30)	304 (24)
70–80	9 (02)	48 (07)	57 (05)
>80	0 (00)	4 (01)	4 (<1)
**Race** a			
Black/African American	148 (25.6)	70 (10.3)	218 (17.3)
Caucasian/White	405 (70.0)	588 (86.1)	993 (78.7)
Asian	3 (<1.0)	7 (01.0)	10 (<1.0)
Other	23 (04.0)	18 (02.6)	41 (03.3)
**Ethnicity**			
Hispanic^b^	56 (09.7)	44 (06.4)	100 (07.9)
**Smoking**			
Ever Smoked MACS Visit 1 to 24 months before HPV testing			
Yes	427 (73.8)	486 (71.2)	913 (72.4)
No	152 (26.2)	197 (28.8)	349 (27.6)
Ever Smoked within last 24 months before HPV testing ^c^			
Yes	189 (32.6)	123 (18.0)	312 (24.7)
No	390 (67.4)	560 (82.0)	950 (75.3)
**AIDS Defining Illnesses^d^**			
None	486 (83.9)	–	–
Kaposi’s Sarcoma	17 (02.9)	–	–
Pneumocystis Pneumonia	26 (04.5)	–	–
Non-Hodgkin’s Lymphoma	1 (00.2)	–	–
Wasting Syndrome	15 (02.6)	–	–
**HPV-DNA Prevalence**			
None	54 (09.3)	203 (29.7)	257 (20.4)
≥*1* HPV Type	525 (90.7)	480 (70.3)	1005 (79.6)
**IARC HPV Carcinogenicity Classification**			
Group 1, strong carcinogens	396 (68)	306 (45)	702 (56)
Group 2, weaker carcinogens	255 (44)	186 (27)	441 (35)
Lower Risk	445 (77)	322 (47)	767 (61)
			
HPV 16 or 18	180 (31.1)	139 (20.4)	319 (25.3)
*HPV 16*	*133 (23.0)*	*109 (15.9)*	*242 (19.2)*
*HPV 18*	*69 (11.9)*	*40 (05.9)*	*109 (08.6)*

	Seropositive		Seronegative		Total	
Characteristic	Mean (SD)	Median	Mean (SD)	Median	Mean (SD)	Median
**Number Sexual Partnerships Reported**						
Lifetime Total at first MACS Visit ^e^	302.(100)	100	244.(90)	90.0	270 (488)	100
MACS Visit 1–24 Months Before the HPV Test Visit^ f^	229 (504)	89	200 (351)	82	213 (428)	84
**Number of Receptive Anal Intercourse Sexual Partnerships Reported**						
During the Last 24 Months Before the HPV Test Visit ^g^	6.1 (19.3)	1	2.9 (7.6)	0	4.4 (14.3)	0
**CD4 T-Cell Characteristics**						
CD4 cell count (cells/mm^3^)	609.(282)	583	929.(310)	900.0	782.(338)	737
Nadir CD4+ T-cell count (cells/mm^3^)	258 (159)	249	–	–	–	–
**Other HIV-infection Characteristics**						
HIV-infection Duration (years)	17.8 (7.6)	18.6	–	–	–	–
Duration CART Use (years)	10.3 (4.4)	12.1	–	–	–	–
Percent of Visits with HIV mRNA <50 copies/mL (%)	52.7 (29.4)	52.9	–	–	–	–

a *p*<0.001, ^b^ *p* = 0.037, ^c^ *p*<0.001, ^d^categories are not mutually exclusive, ^e^ *p* = 0.062; ^f^ *p* = 0.138^ g^ *p*<0.001. P-values for comparing HIV+ vs. HIV- were by the Fisher exact test or the Wilcoxon nonparametric test, as appropriate.

#### Type and group-specific HPV infection characteristics

Most men tested positive for one or more HPVs; however, only 25% (319/1262) tested positive for HPV16 or -18 alone or in combination with other HPVs (Table 1). HIV-infected men showed a higher prevalence of HPV16/18 infection than HIV-uninfected men: 31% (180/579) vs. 20% (139/683) (Table 1). Among hrHPVs, HPV52 (23%, 287/1262), HPV16 (19%, 242/1262), and HPV53 (13%, 169/1262) were most commonly detected while HPV45 (10%, 131/1262) and HPV18 (9%, 109/1262) were less often found (Table S1). The prevalence of viruses with close genetic similarity to HPV16 and HPV18 was high (Table S2). Specifically, 35% (445/1262) of men tested positive for α-7 (HPV18, 39, 45, 59, 68, 70) and 41% (510/1262) tested positive for α-9 (HPV16, 31, 33, 35, 52, 58) phylogenetically-related viruses (Table S2). LrHPVs were detected commonly: HPV6 (16%, 203/1262), followed by HPV61 (14%, 170/1262) and HPV62 (13%, 163/1262) (Table S1). However, only 28% (355/1262) of men tested positive for viruses genetically related to HPV6 or HPV11, α-10 HPV types (HPV6, 11, 44, 55, 74) (Tables S1 & S2).

Group 1 hrHPVs and lrHPVs were detected most often, while Group 2 hrHPVs were less commonly found: 56% (702/1262), 61% (767/1262), and 35% (441/1262), respectively (Table S1). Nearly 20% (250/1262) of men tested positive for a single HPV type, most of which were lrHPVs (Table 2). Additionally, 75% (755/1005) of HPV-infected men tested positive for multiple individual HPV types (multi-type infections), 86% (649/755) of whom tested positive for viruses from >1 IARC-risk category (multi-risk-group infections) (Table 2). HIV-uninfected men tested positive for a solitary HPV 1.8-times more often than HIV-infected men: 25% (167/683) vs. 14% (83/579) (Table 2). The prevalence of simultaneous infection by one or more Group 1 hrHPVs and lrHPVs was greater for HIV-infected than –uninfected men: 62% (327/525) versus 38% (184/480), respectively (*p*<0.05, Table 2). Group 2 hrHPVs were detected infrequently among HPV-infected men in comparison to Group 1 hrHPVs and lrHPVs, both in single-type infections (4% (40/1005), versus 8% (76/1005), and 13% (134/1005), respectively) and as multi-type, single-risk-group infections (<1% (7/1005) versus 5% (51/1005) and 5% (48/1005), respectively). Group 2 infections found in multi-type, multi-risk-group infections were predominantly in combination with both Group 1 hrHPVs and lrHPVs (39%, 255/649), and seldom detected exclusively with Group 1 (10%, 64/649) or lrHPVs (11%, 74/649), alone (Table 2). LrHPVs were detected very often, overall, whether in single-type infections (54%, 134/250), in multi-type, single-risk-group infections (45%, 48/106), or in multi-type, multi-risk-group infections (90%, 585/649; Table 2). Multiple-risk-group, multi-type Group 1, 2, and lower-risk HPV infections were more prevalent among HIV-infected than –uninfected men: 44% (173/398) versus 33% (83/251) (p<0.05, Table 2).

**Table 2:** HPV-Infection Characteristics Among 1005 MACS MSM Showing One or More HPVs for Single and Multiply Detected HPV-Types.

		HIV seropositive(N = 579)	HIV seronegative(N = 683)	Total
**All Men**				
None		54 (09.3)	203 (29.7)	257 (20.4)
*≥1* HPV Type		525 (90.7)	480 (70.3)	1005 (79.6)
**HPV-DNA Positive Men Only**				
		(N = 525) n (%)	(N = 480) n (%)	(N = 1005) n (%)
**Single HPV Detected** a		**83 (15.8)**	**167 (34.8)**	**250 (24.9)**
* Group 1*		*22 (26.5)*	*54 (32.3)*	*76 (31.4)*
* Group 2*		*9 (10.8)*	*31 (18.6)*	*40 (16.0)*
* Low-risk* a		*52 (62.7)*	*82 (49.1)*	*134 (53.6)*
**Multiple HPVs Detected** a		**442 (84.2)**	**313 (65.2)**	**755 (75.1)**
Single HPV Risk-Group^a^		***44 (10.0)***	***62 (19.8)***	***106 (14.0)***
* Group 1*		*20 (45.5* ***)***	*31 (50.0)*	*51 (48.1)*
* Group 2*		*2 (* ***0*** *4.5)*	*5 (* ***0*** *8.1)*	*7 (06.6)*
* Low-risk*		*22 (50.0)*	*26 (41.9)*	*48 (45.3)*
2–3 HPV Risk-Groups^a^		***398 (90.0)***	***251 (80.2)***	***649 (86.0)***
* Group 1 and Group 2* a		*27 (^0^6.8)*	*37 (14.7)*	*64 (^0^9.9)*
* Group 1 and Low-risk*		*154 (38.7)*	*101 (40.2)*	*255 (39.3)*
* Group 2 and Low-risk*		*44 (11.1)*	*30 (12.0)*	*74 (11.4)*
* Group 1, 2, and Low risk* a		*173 (43.5)*	*83 (33.1)*	*256 (39.4)*

a *p*<0.05 Comparing prevalence of infection for HIV+ to HIV-negative men.

#### Age and HPV infections

**Figure 1:**
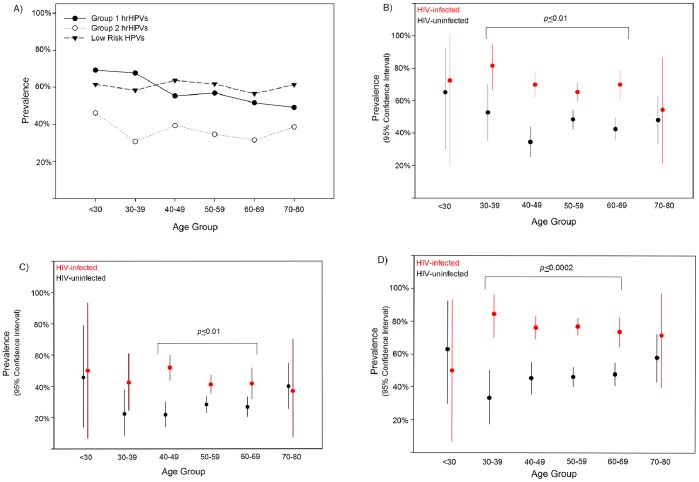
A–D: Age-specific Prevalence of Group 1 and 2 High-risk HPVs and Lower-risk HPVs for 1262 Men Enrolled in the Multicenter AIDS Cohort Study Anal Health Sub-study. (A) Virus Type Group-specific Prevalence for All Men. (B–D) Comparison of 579 HIV-infected and 683–Uninfected Men for (B) Group 1 High-risk HPVs, (C) Group 2 High-risk HPVs, and (D) Lower-risk HPVs.

Anal HPV prevalence estimates were high across the age spectrum (Figure 1A). For men 40 years and older, lrHPV prevalence was highest overall (Figure 1A). For example, 40–69 year old HIV-infected men tested positive for Group 1, 2, and lower-risk HPVs more often than HIV–uninfected men (Figure 1B–D). HIV-infected men as young as 30 years tested positive for Group 1 hrHPVs and lrHPVs more often than HIV-uninfected men (Figure 1D).

HIV-infection characteristics, some sexual behaviors, race and some smoking characteristics only modestly affected the prevalence of Group 1 and 2 hrHPVs and lrHPVs. However, White men showed 1.3-fold lower prevalence of Group 2 HPVs than did minority men (p<0.01) and self-report for tobacco smoking between Visit 1 and the study visit 24 months before HPV testing was positively associated with the prevalence of Group 2 hrHPVs and lrHPVs (PR = 1.21, *p* = 0.04, PR = 1.10 *p* = 0.05). Among HIV-infected men alone, bivariate analyses suggested men with <500 CD4+ cells/mm^3^, a higher lifetime number of sexual partners, and the number of RAI partners during the last 24 months of observation were positively associated with the prevalence of Group 1 hrHPVs (PR: 1.14–1.23, p-values <0.01–0.04). Only CD4+ cell count at the HPV test visit, the number of sex partners and RAI partnerships reported for Visit 1 to the visit 24 months before testing and during the last 24 months, respectively, positively affected Group 2 HPV prevalence estimates (PR: 1.2–1.5, p-values: <0.001–0.02). Men who reported complete adherence to CART during the 4 days preceding the HPV-test visit showed lower prevalence of Group 1 hrHPVs (PR = 0.84, *p*<0.01). Every 10% increase in the total number of HIV+ study visits where HIV-load measured <50 copies/mL was protective for Group 1 and Group 2 hrHPVs, alone in the bivariate analyses, i.e., PR = 0.98, p = 0.03 and PR = 0.89, p<0.0001, respectively.

### Multivariate Analyses

**Figure 2:**
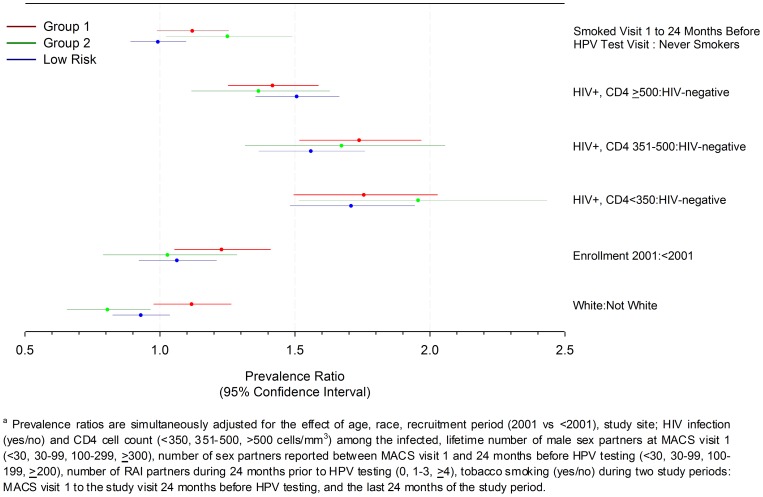
Comparison of Prevalence Ratios for Group 1 and 2 High-risk and Lower-risk HPVs for 1262 Men Group 1 and 2 High-risk HPVs and Lower-risk HPVs for 1262 Men Enrolled in the Multicenter AIDS Cohort Study Anal Health Sub-study.^a^

In separate multivariate analyses for Groups 1, 2 and lower-risk HPVs, data for all men showed that HIV-infection positively affected the prevalence of HPVs detected in anal cytology specimens at all levels of CD4+ cell count (Figure 2). Specifically, the prevalence of Group 1, 2 and lower-risk HPVs was 35–92% higher for HIV-infected men with >500, 350–500 and <350 CD4+ cells/mm^3^ when compared to HIV-uninfected men (*p*-values<0.002, Figure 2). Also, Group 2 hrHPV prevalence alone was 1.2-fold higher for men reporting ever smoking between the first MACS visit and the visit 24 months before HPV testing; conversely, White men also showed nearly 1.3-fold lower prevalence of Group 2 hrHPVs than non-whites (Figure 2). Compared to men enrolled after 2001, those enrolled earlier showed 22% (95% CI: 5%, 41%) higher prevalence for Group 1 hrHPVs (*p*<0.05) alone (Figure 2). Also, the number of sexual partnerships reported across the risk period only modestly influenced the prevalence of HPV infections (Table S3). For example, the prevalence of Group 1, 2 and lower-risk HPVs was 17–35%, on average, for men reporting *≥*4 RAI partners over the 24 months preceding HPV testing (*p*-values<0.005, Table S3). Men reporting *≥*30 lifetime sex partners at their first MACS visit showed 17–24% higher prevalence of lrHPVs than men reporting fewer (Table S3, *p*-values<0.03). Last, the adjusted analyses showed age, self-report for smoking in the 24 months prior to HPV testing, and study site did not influence prevalence of Group 1, 2 or lower-risk HPVs (p-values>0.05).

**Figure 3:**
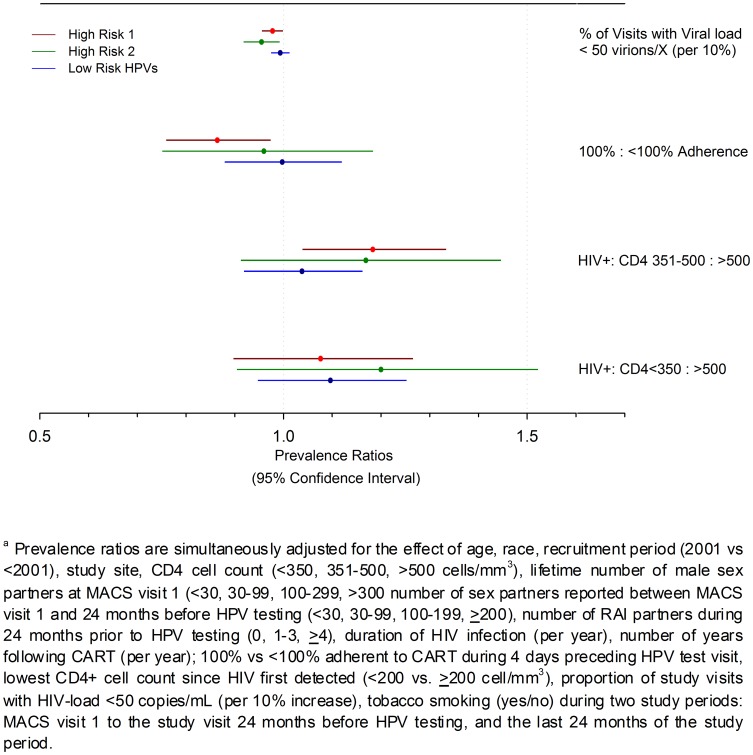
Comparison of Prevalence Ratios for Group 1 and 2 High-risk and Lower-risk HPVs for 579 HIV-infected Men Enrolled in the Multicenter AIDS Cohort Study Anal Health Sub-study.^a^

Multivariate analyses limited to HIV-infected men alone showed adherence to CART treatment, higher CD4+ cell count, and higher proportion of study visits with low HIV-load were statistically significantly associated with the prevalence of Group 1, 2 or lrHPV infections (Figure 3). Specifically, the prevalence of Groups 1 and 2 hrHPVs was 2–5% lower for every 10% increase in the number of HIV+ study visits where HIV-load measured <50 copies mRNA/mL (Figure 3). Men reporting complete adherence to CART for the four days preceding the study visit showed 16% lower prevalence of Group 1 hrHPVs, alone, in comparison to less adherent men: PR = 0.86 (0.76, 0.97) (Figure 2). Additionally, the prevalence of Group 1 hrHPVs alone was 17% higher for men showing 350–500 CD4+ cells/mm^3^, compared to men with >500 T-lymphocytes/mm^3^ (*p* = 0.01, Figure 2). Among HIV-infected men, sexual partnerships again affected prevalence of all virus groupings modestly. Although prevalence of Group1 hrHPVs and lrHPVs was unaffected, the prevalence of Group 2 hrHPVs alone was nearly 1.4 higher for men reporting *≥*4 RAI partners during the 24 months before HPV testing than men reporting none (Table S4). Lifetime number of sexual partners only affected the prevalence of lrHPVs, albeit modestly, with men reporting *≥*30 partners showing 14–18% higher prevalence of infection over men reporting <30 partners (p-values <0.05 for 1 of 3 comparisons; Table S4). Age, race, enrollment period, total HIV-infection duration, years of CART use, tobacco smoking, and study site did not influence prevalence estimates for Group 1, 2 or lower-risk HPVs in the multivariate adjusted analyses (p-values>0.05).

## Discussion

This study demonstrates that HPV infections are common, and are associated with age and HIV-infection. Specifically, HIV-infected men 40–69 years of age are at higher risk for Group 1, 2 and lower-risk HPVs than –uninfected men. However, among the youngest and oldest men evaluated, we observed no statistically significant differences. Expectedly, our data suggest HPV infection prevalence is high among young adult MSM, largely irrespective of HIV-serostatus. These findings are consistent with reports by others that suggest the prevalence of any anal HPV infection among HIV-infected MSM may range from 30% to >90% [32]–[35]. However, some reports suggest the prevalence of anal HPV infections for men who have sex with women may range from 0–33%, compared to studies that show prevalence estimates as high as 68% among HIV-infected MSM [20], [21], [36]–[38].

Most important, the prevalence of strongly and weakly carcinogenic HPVs is unequal among HIV-infected and -uninfected men. The prevalence of anal Group 1 hrHPVs and lrHPVs were similarly high across the age spectrum, while Group 2 hrHPVs were detected less frequently. Comparatively, prevalence estimates for a large sample of HIV-uninfected U.S. MSM showed a similar flat, albeit lower, age-specific prevalence of lr- and hrHPVs than reported herein [23]. Global age-specific prevalence patterns for women differ sharply, with peak prevalence estimates among females under 25, diminishing thereafter through their early 40 s, from ∼20% to ∼10%, overall, with some regional variation [5]. However, one tri-national study of 176 MSM reported an inverse association between age and anal HPV-prevalence, overall (∼60% to <40%), and for hrHPVs (∼40% to <20%) over a similar age span [24].

Finding a high prevalence of Group 1 hrHPVs in anal specimens from HIV-infected MSM is important for cancer prevention strategies. A recent report shows HIV-infected anal cancer cases evidence 2.3–4.5 times higher odds than matched control for detecting anti-HPV16, 18, 31, 33, 35, 45, 52 or 58 L1 antibodies (p-values:<0.05) in serum, all Group 1 hrHPVs [39]. In addition, the temporal analyses of these Swiss HIV Cohort data show anal cancer cases evidenced 5.9–14–fold (p-values:≤ 0.05) higher odds of showing <350 CD4+ cells/mm^3^ as early as 6–7 years before diagnosis than did age-, region-, and transmission mode-matched controls, but no statistically significant higher odds related to HIV-load [39]. Consistent to these reports, our data show HIV-load <50 copies/mL protects against Group 1 and 2 hrHPV infections and that complete adherence to CART protects against Group 1 hrHPVs, as well. Important to our findings, experts conclude that even moderate HIV-related immune suppression measured as many as 6–7 years before an anal cancer diagnosis suggest that better HIV-treatment adherence may decrease risk for persistent Group 1 hrHPV infections that likely cause most anal cancers [40].

Our findings showed that the prevalence of Group 2 hrHPVs was lower among Whites than non-Whites, suggesting that sexual network characteristics may vary by race. To date, we find no studies that evaluated the prevalence of these *possible-probable* weaker carcinogens separately from strongly carcinogenic hrHPVs. Generally, published data for women suggest that prevalence of HPV-infections varies geographically, among those with normal and abnormal cytology and for related cancers [5], [41]–[43]. Few data [44] suggest that HPV-infection prevalence within geographic regions varies by race [41]–[43]. Neither Nyitray et al. or Chin-Hong and colleagues report an effect of race in their multivariate analyses [23], [24]. These high prevalence trends, especially among older MSM, are important for cancer prevention, surveillance, and program planning. The proportion of anal cancer cases who are infected with HIV has risen dramatically and the age at diagnosis among HIV-infected men may be trending younger since the introduction of effective antiretroviral therapies for HIV infection [45], [46]. Thus, our finding that Group 2 HPV prevalence was less common among Whites than men of other races suggests it is important to monitor type-specific trends in HPV-related anal cancers as infection with Group 2 carcinogenic HPVs may differ by race.

Tobacco smoking is long associated with uterine cervical HPV-infections and –related cancers in women [47], [48]; however, recently published findings are mixed for anal HPV infections. For example, adult HIV-infected anal cancer cases show 2.6-fold higher odds of current tobacco use when compared to matched controls [39]. Nielson et al., reported current tobacco users showed 2.1-fold higher odds (p<0.05) than never smokers for detecting any hrHPV on external genital or internal anal surfaces [49]; in contrast, we found higher risk for detection of Group 2 hrHPVs alone when smokers and non-smokers were compared. However, Nyitray et al., report the odds of detecting any HPVs and hrHPVs among HIV-uninfected MSM may be mixed: 1.2 and 0.8 (p>0.05) respectively [24].

These analyses may be limited. Few very young and few men over 70 years old may limit our comparisons for age-specific infection prevalence, and incident and persistent infections cannot be discerned using cross-sectional data. While the assay detected all Group 1 hrHPVs, we were unable to evaluate four Group 2 hrHPVs (i.e., HPV30, 34, 85, and 97) [14]. Also, MACS data cannot discriminate between men reporting the same partner at every visit from men reporting a new single sex partner at each study visit. While most variables analyzed herein were informed objective criteria and laboratory tests, some relied upon self-report data that may be misreported, especially where topics are socially sensitive. The direction of bias introduced by misclassified polychotomous or interrelated variables may be unpredictable [50], [51]. Last, passage of a sterile Dacron swab through the anal verge cannot completely discriminate between HPVs on external anogenital surfaces and intra-anal infections alone.

The prevalence of high-risk Group 1 HPVs, that include HPV16, may explain escalating IAC rates among HIV-infected men over the past decade [1], [2], [12], [39]. Our analyses support strategies that seek to increase HIV-medication compliance and promote lower HIV-load characteristics among HIV-infected MSM so as to lower risk for most hrHPVs, even if the reduction is modest. Also, our data support prevention messages that promote safe-sex practices, including long-term mutual monogamy and condom use to prevent exposure to new hrHPVs. Last, the high prevalence of multi-type and multi-group HPV infections in these men warrants further study to determine whether HIV and multi-type HPV infections synergistically act to increase anal cancer risk. Overall, our analyses warrant careful surveillance for Group 1 and 2 HPVs using tumor registry data and ongoing longitudinal studies of high-risk MSM.

## Supporting Information

Table S1 **Prevalence of Type-specific HPV-DNA detected in residual anal cytology specimens for 1262 MSM.**
(DOCX)

Table S2 **Frequency and Proportional Distribution of HPV Phylogenetic Family Classification Characteristics for Residual Anal Cytology Specimens Obtained from 1262 MACS Participants.**
(DOCX)

Table S3 **Comparison of the Effect of Self-reported Sexual Partnerships for Three Temporal Periods on the Prevalence of Group 1, 2, High-risk and Lower risk HPVs Detected in Anal Cytology Specimens Gathered from 1262 Multicenter AIDS Cohort Study Men.** (Prevalence Ratios and 95% Confidence Estimates).
(DOCX)

Table S4 **Comparison of Effects of Self-reported Sexual Partnerships Reported over Three Temporal Periods on the Prevalence of Group 1, 2, High-risk and Lower risk HPVs Detected in Anal Cytology Specimens Gathered from 579 HIV-infected Multicenter AIDS Cohort Study Men.** (Prevalence Ratios and 95% Confidence Estimates).
(DOCX)
