# Supplementary material for: Factors Affecting the Prevalence of Strongly and Weakly Carcinogenic and Lower-Risk Human Papillomaviruses in Anal Specimens in a Cohort of Men Who Have Sex with Men (MSM)
Source: PLoS One. 2013 Nov 20;8(11):e79492. doi: 10.1371/journal.pone.0079492 (PMC3835810; doi:10.1371/journal.pone.0079492)
Supplement: Table S1 — Prevalence of Type-specific HPV-DNA detected in residual anal cytology specimens for 1262 MSM. (DOCX) [file pone.0079492.s001.docx]

Table S1: Prevalence of Type-specific HPV-DNA detected in residual anal cytology specimens for 1262 MSM.

| HPV *Type* | HIV-infected (N=579) | HIV-uninfected (N=683) | Total  (N=1262) |
| --- | --- | --- | --- |
|  | n (%) | n (%) | n (%) |
| Negative for all 37 HPVs |  |  |  |
| Group 1 |  |  |  |
| **HPV16** | 133 (23.0) | 109 (16.0) | 242 (19.2) |
| **HPV18** | 69 (11.9) | 40 (05.9) | 109 (08.6) |
| **HPV31** | 54 (09.3) | 47 (06.9) | 101 (08.0) |
| **HPV33** | 46 (07.9) | 22 (03.2) | 68 (05.4) |
| **HPV35** | 62 (10.7) | 16 (02.3) | 78 (06.2) |
| **HPV39** | 47 (08.1) | 40 (05.9) | 87 (06.9) |
| **HPV45** | 88 (15.2) | 43 (06.3) | 131 (10.4) |
| **HPV51** | 66 (11.4) | 41 (06.0) | 107 (08.5) |
| **HPV52** | 191 (33.0) | 96 (14.1) | 287 (22.7) |
| **HPV56** | 40 (06.9) | 29 (04.3) | 69 (05.5) |
| **HPV58** | 82 (14.2) | 36 (05.3) | 118 (09.4) |
| **HPV59** | 67 (11.6) | 31 (04.5) | 98 (07.8) |
| Group 2a & 2b |  |  |  |
| HPV26 | 7 (01.2) | 3 (00.4) | 10 (00.8) |
| HPV53 | 99 (17.1) | 70 (10.2) | 169 (13.4) |
| HPV66 | 52 (09.0) | 32 (04.7) | 84 (06.7) |
| HPV67 | 13 (02.3) | 10 (01.5) | 23 (01.8) |
| **HPV68** | 44 (07.6) | 35 (05.1) | 79 (06.3) |
| **HPV69** | 14 (02.4) | 9 (01.3) | 23 (01.8) |
| HPV70 | 76 (13.1) | 41 (06.0) | 117 (09.3) |
| HPV73 | 33 (05.7) | 25 (03.7) | 58 (04.6) |
| HPV82 | 16 (02.8) | 10 (01.5) | 26 (02.1) |
| Lower Risk HPVs |  |  |  |
| HPV6 | 128 (22.1) | 75 (11.0) | 203 (16.1) |
| HPV11 | 40 (06.9) | 21 (03.1) | 61 (04.8) |
| HPV40 | 7 (01.2) | 8 (01.2) | 15 (01.2) |
| HPV42 | 55 (09.5) | 33 (04.8) | 88 (07.0) |
| HPV54 | 67 (11.6) | 29 (04.3) | 96 (07.7) |
| HPV55 | 104 (18.0) | 42 (06.2) | 146 (11.6) |
| HPV61 | 109 (18.8) | 61 (08.9) | 170 (13.5) |
| HPV62 | 104 (18.0) | 59 (08.6) | 163 (12.9) |
| HPV64 | 4 (00.7) | 1 (00.2) | 5 (00.4) |
| HPV71 | 8 (01.4) | 5 (00.7) | 13 (01.0) |
| HPV72 | 56 (09.7) | 30 (04.4) | 86 (06.8) |
| HPV81 | 58 (10.0) | 17 (02.5) | 75 (05.9) |
| HPV83 | 41 (07.1) | 22 (03.2) | 63 (05.0) |
| HPV84 | 78 (13.5) | 46 (06.7) | 124 (09.8) |
| IS39 | 12 (02.1) | 5 (0.7) | 17 (01.4) |
| CP6108 | 73 (12.6) | 54 (7.9) | 127 (10.1) |
