# Supplementary material for: Factors Affecting the Prevalence of Strongly and Weakly Carcinogenic and Lower-Risk Human Papillomaviruses in Anal Specimens in a Cohort of Men Who Have Sex with Men (MSM)
Source: PLoS One. 2013 Nov 20;8(11):e79492. doi: 10.1371/journal.pone.0079492 (PMC3835810; doi:10.1371/journal.pone.0079492)
Supplement: Table S2 — Frequency and Proportional Distribution of HPV Phylogenetic Family Classification Characteristics for Residual Anal Cytology Specimens Obtained from 1262 MACS Participants. (DOCX) [file pone.0079492.s002.docx]

Table S2: Frequency and Proportional Distribution of HPV Phylogenetic Family Classification Characteristics for Residual Anal Cytology Specimens Obtained from 1262 MACS Participants

| Phylogenetic Virus Families | HIV-infected (N=579) | HIV-uninfected (N=683) | Total  (N=1252) |
| --- | --- | --- | --- |
|  | n (%) | n (%) | n (%) |
| High-risk |  |  |  |
| α-7 (HPV18, 39, 45, 59, 68, 70) | 264 (45.6) | 181 (26.5) | 445 (35.3) |
| α-9 (HPV16, 31, 33, 35, 52, 58) | 301 (52.0) | 209 (30.6) | 510 (40.4) |
| α-5 (HPV26, 51, 69, 82, 83, IS39) | 100 (17.3) | 62 (9.1) | 162 (12.8) |
| α-6 (HPV53, 56, 66) | 166 (28.7) | 121 (17.7) | 287 (22.7) |
| Low Risk |  |  |  |
| α-10 (HPV6, 11, 44, 55, 74) | 226 (39.0) | 129 (18.9) | 355 (28.1) |
| α-3 (HPV61, 62, 72, 81, 83, 84, 89) | 302 (52.2) | 189 (27.7) | 491 (38.9) |
