# Supplementary material for: Factors Affecting the Prevalence of Strongly and Weakly Carcinogenic and Lower-Risk Human Papillomaviruses in Anal Specimens in a Cohort of Men Who Have Sex with Men (MSM)
Source: PLoS One. 2013 Nov 20;8(11):e79492. doi: 10.1371/journal.pone.0079492 (PMC3835810; doi:10.1371/journal.pone.0079492)
Supplement: Table S3 — Comparison of the Effect of Self-reported Sexual Partnerships for Three Temporal Periods on the Prevalence of Group 1, 2, High-risk and Lower risk HPVs Detected in Anal Cytology Specimens Gathered from 1262 Multicenter AIDS Cohort Study Men. (Prevalence Ratios and 95% Confidence Estimates). (DOCX) [file pone.0079492.s003.docx]

Table S3: Comparison of the Effect of Self-reported Sexual Partnerships for Three Temporal Periods on the Prevalence of Group 1, 2, High-risk and Lower risk HPVs Detected in Anal Cytology Specimens Gathered from 1262 Multicenter AIDS Cohort Study Men. (Prevalence Ratios and 95% Confidence Estimates)

|  | International Agency for Research on Cancer Risk Classification Categories for *Human papillomaviruses* (HPVs) | | |
| --- | --- | --- | --- |
|  | High-Risk HPVs | |  |
|  | Group 1  (95% CI)^a^ | Group 2  (95% CI) ^a^ | Lower-risk HPVs  (95% CI) ^a^ |
| Number of Anal Receptive Intercourse Partners Reported over the 24 Months Preceding HPV Testing | | | |
| 4+ versus 0 | **1.23 (1.09, 1.39)** | **1.35 (1.11, 1.63)** | **1.17 (1.05, 1.30)** |
| 1-3 versus 0 | **1.17 (1.03, 1.32)** | 1.17 (0.96, 1.44) | **1.13 (1.01, 1.26)** |
| Number of Partners Reported between MACS Visit 1 and the Visit 24 Months Preceding HPV Testing | | | |
| >200 versus <30 | 1.19 (0.99, 1.43) | 1.26 (0.93, 1.69) | **1.22 (1.03, 1.44)** |
| 100-199 versus <30 | **1.21 (1.02, 1.44)** | 1.13 (0.84, 1.51) | 1.14 (0.98, 1.34) |
| 30-99 versus <30 | 1.13 (0.97, 1.33) | 1.11 (0.85, 1.43) | 1.05 (0.91, 1.22) |
| Number of Lifetime Partners Reported at the First MACS Study Visit | | | |
| >300 versus <30 | 1.11 (0.94, 1.31) | 1.27 (0.98, 1.65) | **1.23 (1.06, 1.43)** |
| 100-299 versus <30 | 1.16 (0.99, 1.36) | 1.08 (0.85, 1.41) | **1.17 (1.01, 1.35)** |
| 30-99 versus <30 | **1.17 (1.00, 1.36)** | 1.16 (0.91, 1.48) | **1.21 (1.05, 1.39)** |

^a^ Prevalence ratios are simultaneously adjusted for the effect of age, race, recruitment period (2001 vs <2001), study site; HIV infection (yes/no) and CD4 cell count (<350, 351-500, >500 cells/mm^3^) among the infected, lifetime number of male sex partners at MACS visit 1 (<30, 30-99, 100-299, >300), number of sex partners reported between MACS visit 1 and 24 months before HPV testing (<30, 30-99, 100-199, >200), number of RAI partners during 24 months prior to HPV testing (0, 1-3, >4), tobacco smoking (yes/no) during two study periods: MACS visit 1 to the study visit 24 months before HPV testing, and the last 24 months of the study period.
