# Supplementary material for: Factors Affecting the Prevalence of Strongly and Weakly Carcinogenic and Lower-Risk Human Papillomaviruses in Anal Specimens in a Cohort of Men Who Have Sex with Men (MSM)
Source: PLoS One. 2013 Nov 20;8(11):e79492. doi: 10.1371/journal.pone.0079492 (PMC3835810; doi:10.1371/journal.pone.0079492)
Supplement: Table S4 — Comparison of Effects of Self-reported Sexual Partnerships Reported over Three Temporal Periods on the Prevalence of Group 1, 2, High-risk and Lower risk HPVs Detected in Anal Cytology Specimens Gathered from 579 HIV-infected Multicenter AIDS Cohort Study Men. (Prevalence Ratios and 95% Confidence Estimates). (DOCX) [file pone.0079492.s004.docx]

Table S4: Comparison of Effects of Self-reported Sexual Partnerships Reported over Three Temporal Periods on the Prevalence of Group 1, 2, High-risk and Lower risk HPVs Detected in Anal Cytology Specimens Gathered from 579 HIV-infected Multicenter AIDS Cohort Study Men. (Prevalence Ratios and 95% Confidence Estimates)

|  | International Agency for Research on Cancer Risk Classification Categories for 37 *Human papillomaviruses* (HPVs) | | |
| --- | --- | --- | --- |
|  | High Risk HPVs | |  |
|  | Group 1  (95% CI)^a^ | Group 2  (95% CI) ^a^ | Lower-risk HPVs  (95% CI) ^a^ |
| Number of Anal Receptive Intercourse Partners Reported over the 24 Months Preceding HPV Testing | | | |
| 4+ versus 0 | **1.15 (1.00, 1.33)** | **1.32 (1.03, 1.68)** | 1.06 (0.94, 1.20) |
| 1-3 versus 0 | 1.13 (0.97, 1.32) | 1.23 (0.94, 1.60) | 1.10 (0.97, 1.24) |
| Number of Partners Reported between MACS Visit 1 and the Visit 24 Months Preceding HPV Testing | | | |
| >200 versus <30 | 1.14 (0.93, 1.40) | 1.33 (0.91, 1.94) | 1.07 (0.90, 1.27) |
| 100-199 versus <30 | 1.05 (0.86, 1.29) | 1.04 (0.71, 1.53) | 0.97 (0.82, 1.15) |
| 30-99 versus <30 | 1.04 (0.86, 1.25) | 1.15 (0.83, 1.59) | 0.92 (0.78, 1.07) |
| Number of Lifetime Partners Reported at the First MACS Study Visit | | | |
| >300 versus <30 | 1.09 (0.90, 1.33) | 1.00 (0.71, 1.40) | **1.17 (1.00, 1.38)** |
| 100-299 versus <30 | 1.15 (0.96, 1.38) | 0.94 (0.67, 1.30) | 1.14 (0.97, 1.34) |
| 30-99 versus <30 | 1.11 (0.92, 1.34) | 0.95 (0.69, 1.30) | **1.19 (1.02, 1.39)** |

^a^ Prevalence ratios are simultaneously adjusted for the effect of age, race, recruitment period (2001 vs <2001), study site, CD4 cell count (<350, 351-500, >500 cells/mm^3^), lifetime number of male sex partners at MACS visit 1 (<30, 30-99, 100-299, >300 number of sex partners reported between MACS visit 1 and 24 months before HPV testing (<30, 30-99, 100-199, >200), number of RAI partners during 24 months prior to HPV testing (0, 1-3, >4), duration of HIV infection (per year), number of years following CART (per year); 100% vs <100% adherent to CART during 4 days preceding HPV test visit, lowest CD4+ cell count since HIV first detected (<200 vs. >200 cell/mm^3^), proportion of study visits with HIV-load <50 copies/mL (per 10% increase), tobacco smoking (yes/no) during two study periods: MACS visit 1 to the study visit 24 months before HPV testing, and the last 24 months of the study period.
